# Protective Effects of Oleanolic Acid on Human Keratinocytes: A Defense Against Exogenous Damage

**DOI:** 10.3390/ph18020238

**Published:** 2025-02-11

**Authors:** Marzia Vasarri, Maria Camilla Bergonzi, Manuela Leri, Rebecca Castellacci, Monica Bucciantini, Lucia De Marchi, Donatella Degl’Innocenti

**Affiliations:** 1 Department of Experimental and Clinical Biomedical Sciences, University of Florence, Viale Morgagni 50, 50134 Florence, Italy; marzia.vasarri@unifi.it (M.V.); manuela.leri@unifi.it (M.L.); monica.bucciantini@unifi.it (M.B.); 2 Department of Chemistry “Ugo Schiff”, University of Florence, Via Ugo Schiff 6, Sesto Fiorentino, 50019 Florence, Italy; mc.bergonzi@unifi.it (M.C.B.); rebecca.castellacci@unifi.it (R.C.); 3 Veterinary Teaching Hospital, Department of Veterinary Sciences, University of Pisa, 56124 Pisa, Italy; lucia.demarchi@unipi.it

**Keywords:** oleanolic acid, HaCaT cells, oxidative stress, tert-butyl-hydroperoxide

## Abstract

**Background/objectives:** Aging leads to increased oxidative stress and chronic inflammation in the skin, which contribute to various disorders such as dermatitis and cancer. This study explores the cytoprotective effects of oleanolic acid (OA), a natural triterpenoid compound known for its potential in mitigating oxidative damage, on human keratinocyte (HaCaT) cells exposed to oxidative stress from tert-butyl hydroperoxide (tBHP). **Methods:** Using in vitro experiments, we assessed cell viability, reactive oxygen species (ROS) levels, nitric oxide (NO) production, and protein expression following OA pre-treatment. Advanced imaging techniques were employed to visualize protein localization. **Results:** Results demonstrated that OA significantly improved cell viability and reduced intracellular ROS levels compared with those in controls. Additionally, OA inhibited inducible nitric oxide synthase (iNOS) expression and subsequent nitric oxide release, indicating a modulation of inflammatory responses. Notably, while tBHP activated the Nrf2/HO-1 signaling pathway, OA did not enhance this response, suggesting that OA exerts cytoprotective effects through mechanisms independent of Nrf2 activation. **Conclusion:** OA shows promise in protecting HaCaT cells from tBHP-induced oxidative stress, highlighting its potential role in promoting skin health and addressing aging-related damage. The study proposes that OA operates through pathways distinct from Nrf2 and MAPKs, paving the way for new therapeutic strategies aimed at improving skin health against oxidative stress.

## 1. Introduction

Aging represents a complex biological phenomenon marked by progressive cellular dysfunction and heightened susceptibility to a variety of diseases. This decline is particularly evident in the skin, a vital organ that not only acts as a protective barrier but also undergoes significant age-related changes [1]. The skin aging processes are determined both by chronological aging, which affects the entire body, and by foreign factors to which the skin is subjected, i.e., UV radiation, chemicals, pollution, etc. [2].

Key among these age-related skin changes are increased oxidative stress and chronic inflammation, which can detrimentally alter skin structure and function and contribute to a higher incidence of skin disorders, including dermatitis and skin cancers. The interaction between oxidative stress and inflammation represents an area of growing interest in scientific research, as it highlights how these two phenomena are interconnected and contribute to the onset of numerous age-related disorders. This synergistic link creates a vicious cycle, in which the accumulation of reactive oxygen species (ROS) can cause cellular and tissue damage, which, in turn, activates inflammatory responses that may further exacerbate oxidative stress. Therefore, it is urgent to promote practical and effective interventions capable of significantly altering the course of these conditions [3,4].

Oxidative stress arises when there is an imbalance between the generation of ROS and the cellular antioxidative defenses. Free radicals, produced as byproducts of normal metabolic processes, can be exacerbated by environmental factors such as pollution and UV radiation [5,6]. The accumulation of ROS can result in damage to critical cellular structures, i.e., DNA, proteins, and lipids, disrupting homeostasis and compromising cellular function. As such, elucidating strategies to counteract and prevent oxidative stress is crucial for maintaining skin health and mitigating the repercussions associated with the skin aging process [7,8].

A pivotal regulator of the cellular response to oxidative stress is nuclear factor erythroid 2-related factor 2 (Nrf2). The Nrf2 signaling system has emerged as perhaps the most important cellular defense and survival pathway against oxidative stress and toxicants. Disruption of Nrf2 signaling is associated with an increased susceptibility to oxidative insults [9]. Nrf2 modulates the expression of a multitude of genes that play essential roles in antioxidative defense. Under physiological conditions, Nrf2 is kept at low levels through proteasomal degradation; however, oxidative stress prompts its translocation to the nucleus, where it engages antioxidative response elements (ARE) to activate protective genes. A particularly important player in this process is heme oxygenase-1 (HO-1), whose expression is induced by Nrf2 and which contributes to cellular detoxification and redox homeostasis. Furthermore, the activation of Nrf2 involves intricate signaling pathways, including the mitogen-activated protein kinase (MAPK) cascade, indicative of a multifaceted cellular response to oxidative stimuli [9,10].

Antioxidants are protective molecules against ROS toxicity; while cells produce antioxidative enzymes and metabolites, to neutralize ROS, numerous natural compounds from various sources also play a vital role in maintaining cellular homeostasis. For this reason, the quest for natural compounds with notable antioxidative potential has led to the identification of various plant species rich in bioactive metabolites, such as carotenoids, polyphenols, and essential oils. These secondary plant metabolites possess diverse therapeutic properties, primarily attributed to their antioxidative activities, which play a crucial role in mitigating oxidative stress and its associated detrimental effects [11].

Among these bioactive compounds, oleanolic acid (OA), a natural triterpenoid, has garnered substantial interest in pharmacological research due to its presence in multiple medicinal plants, including *Olea europaea* and *Viscum album* L. [12]. OA is widely recognized for its potent antioxidative and anti-inflammatory properties. Our previous studies have highlighted a significant anti-inflammatory role for OA in murine macrophage cell models [13], as well as its antiglycation activity in vitro [14], thereby underscoring its healing potential in various inflammation- and glycation-related diseases. It is well-known that the aging process, which is influenced by both physiological factors and external agents, is marked by an inflammatory response, along with the glycation of extracellular matrix proteins such as collagen, which contributes to the deterioration of skin health [15,16]. The already recognized properties of OA position it as a promising candidate for addressing the oxidative stress effects associated with aging, particularly regarding skin health. In light of these considerations, this study aims to deepen the understanding of the potential cytoprotective effects of OA on human keratinocyte (HaCaT) cells in the context of oxidative stress induced by the pro-oxidant tert-butyl hydroperoxide (tBHP). HaCaT cells serve as a well-established model for investigating epidermal homeostasis and the mechanisms underlying skin pathophysiology [17].

Existing literature indicates that tBHP can induce oxidative damage to keratinocytes both in vitro and in vivo [18]. Tert-butyl hydroperoxide, an organic peroxide that acts as pro-oxidant, undergoes metabolic conversion in cells via cytochrome P450, leading to the generation of peroxyl and alkoxyl radicals or its detoxification to tert-butanol, both of which contribute to the rapid oxidation and depletion of cellular glutathione. Such pathways are associated with oxidative injury to cells, establishing tBHP as a dependable model for simulating oxidative stress that parallels the damaging effects of environmental stressors, including UV radiation [19].

Our research aims to analyze intracellular ROS production and to examine the expression of inducible nitric oxide synthase (iNOS), with a particular focus on nitric oxide (NO) production levels. Such analyses are fundamental for understanding the cellular mechanisms and possible signaling pathways through which OA exerts its protective actions. The data obtained from this study hold significant importance and could provide insights for further research aimed at preventing and mitigating oxidative damage, thereby contributing to improved skin health.

## 2. Results

### 2.1. The Impact of Oleanolic Acid (OA) on Viability and Oxidative Stress in HaCaT Cells

**Figure 1:**
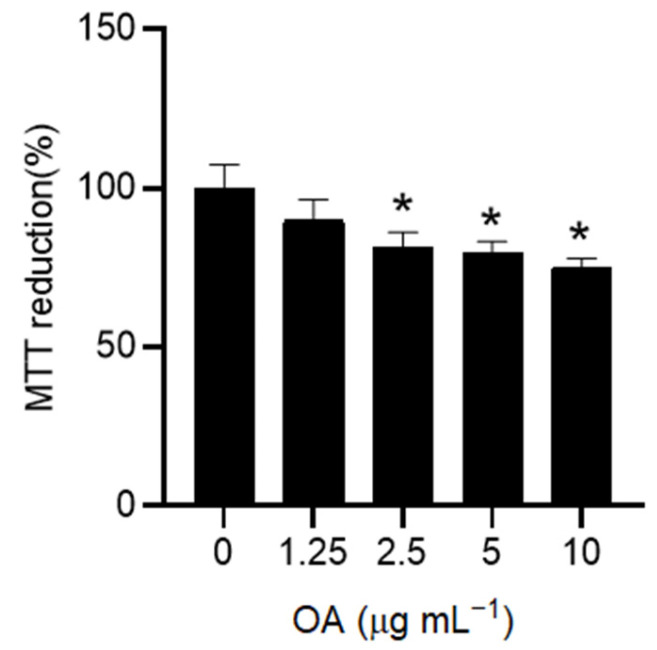
Effect of OA on HaCaT cell viability. HaCaT cells were exposed to increasing doses of OA (0–10 µg mL^−1^) in a serum-free medium for 24 h. Viable cells were detected as metabolically active cells using the MTT assay. Results are expressed as the mean ± standard deviation (SD) of three independent experiments. Statistical analysis was performed by one-way ANOVA followed by the post hoc Tukey’s HSD test (*n* = 3). * *p* < 0.05 compared with untreated control cells (0).

In the present study, the effect of different concentrations of OA was analyzed in HaCaT cells using an MTT assay to evaluate mitochondrial activity as a measure of cell viability. OA exerted a dose-dependent effect at the tested concentrations, ranging from 0 to 10 µg mL^−1^, in accordance with previous results reported in the literature [13] (Figure 1). Based on the outcomes obtained, subsequent experiments were performed using the lowest non-toxic concentration for the cells (1.25 µg mL^−1^ OA) in order to ensure an optimal cellular environment and to minimize potential adverse effects.

**Figure 2:**
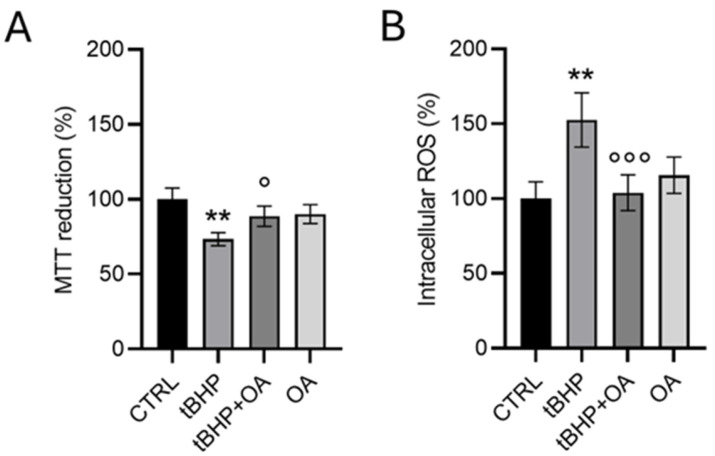
Effect of OA on the viability (**A**) and intracellular ROS formation (**B**) in HaCaT cells exposed to the pro-oxidative agent tBHP. HaCaT cells were pre-treated with OA (1.25 µg mL^−1^) for 2 h and then exposed to tBHP (200 µM) for 3 h in serum-free medium. Untreated cells or those treated with OA or tBHP alone were used as controls. Data are expressed as the mean ± SD of three separate experiments. Statistical analysis was performed using one-way ANOVA followed by Tukey’s HSD test (*n* = 3): ** *p* < 0.01 vs. CTRL; ° *p* < 0.05, °°° *p* < 0.001 vs. tBHP-treated cells.

To simulate an exogenous oxidative insult, HaCaT cells were exposed to the pro-oxidative action of tBHP. The exposure of HaCaT cells to tBHP showed a significant negative impact on cell viability. As shown in Figure 2A, treatment with tBHP (200 µM) resulted in a reduction in cell viability of approximately 30% (73 ± 4%) compared with untreated control cells (CTRL). However, cell pre-treatment with OA (1.25 µg mL^−1^) showed a significant protective effect. In particular, OA pre-treated cells exposed to tBHP exhibited a limited reduction in cell viability of only 12% (88 ± 6%) compared with CTRL, indicating effective protection by OA against the toxicity induced by the pro-oxidative factor. This defensive mechanism induced by OA was further highlighted by intracellular ROS detection. Indeed, treatment with tBHP caused a significant increase in intracellular ROS, with a variation of 50% (152 ± 18%) compared with CTRL. However, OA cell pre-treatment prevented this increase, maintaining comparable intracellular ROS levels to those of untreated control cells, even in the presence of tBHP.

These results suggest that OA pre-treatment triggers an effective protective mechanism against tBHP-induced toxicity in HaCaT cells, mitigating both the reduction in cell viability and the increase in intracellular ROS levels.

### 2.2. The Role of OA in the Expression of iNOS and NO Release

In the skin, the NO derived from the inducible isoform of NOS (iNOS) plays a crucial role in the inflammatory process. Aberrant iNOS activity and NO production in response to inflammatory signals or cellular stress have been implicated in several skin pathologies [20].

**Figure 3:**
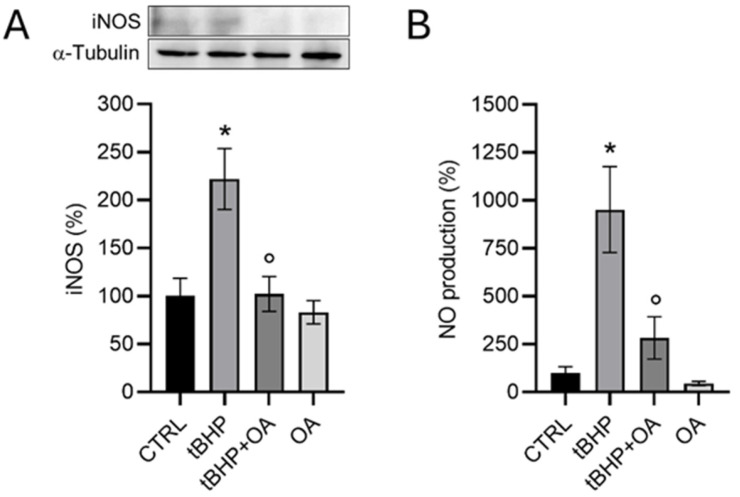
Analysis of iNOS expression by Western blot analysis (**A**) and NO production and release into the culture medium by the Griess test (**B**) in HaCaT cells exposed to the pro-oxidant tBHP. HaCaT cells were pre-treated with OA (1.25 µg mL^−1^) for 2 h and then exposed to tBHP (200 µM) for 3 h in serum-free medium. Untreated cells or cells treated with OA or tBHP alone were used as controls. Data are reported as the mean ± SD of three different experiments. For the Western blot analysis, statistics were performed using the Kruskal–Wallis test followed by Conover’s post hoc test (*n* = 3): * *p* < 0.05 vs. CTRL; ° *p* < 0.05 vs. tBHP-treated cells. For the Griess test, statistics were performed by using one-way ANOVA followed by the post hoc Tukey HSD test (*n* =3): * *p* < 0.05 vs. CTRL; ° *p* < 0.05 vs. tBHP-treated cells.

Western blotting analysis of the inducible isoform of NOS expression in HaCaT cells exposed to the pro-oxidant tBHP revealed a significant increase (222 ± 31%) in iNOS levels compared with control cells (Figure 3A), confirming what has already been suggested in the literature about the activation of inflammatory responses or cellular stress under exposure conditions. Consequently, the pro-oxidative activity of tBHP led to a significant increase in NO synthesis (952 ± 224%), as assessed by the Griess test (Figure 3B).

An interesting aspect that emerged from this research is the protective role of OA. Indeed, OA-pretreatment was able to prevent the increase in iNOS expression levels induced by tBHP. In fact, the levels of iNOS in HaCaT cells pre-treated with OA and subsequently exposed to tBHP were comparable to those of untreated control cells, indicating that OA pre-treatment effectively inhibits the activation of iNOS expression (Figure 3A). This preventive effect of OA on iNOS expression levels translated into reduced synthesis and, therefore, limited release of NO into the culture medium. Indeed, cells pre-treated with OA before exposure to tBHP showed a significant reduction in NO levels, amounting to only 283 ± 109% compared with cells exposed to the pro-oxidative factor alone (Figure 3B). These results suggest a significant modulation of iNOS activity by OA, highlighting a potential protective effect against oxidative stress.

### 2.3. The Role of OA in Modulating the Nrf2/HO-1 Signaling Pathway

The nuclear factor erythroid-2 related factor 2 (Nrf2)/heme oxygenase (HO-1) signaling pathway is an essential signaling pathway in the oxidative stress response [21]. Under physiological conditions, Nrf2 levels are generally low, but in response to pro-oxidative stimuli, they increase and translocate to the nucleus, activating the expression of over 200 protective genes against oxidative stress, including HO-1 [22].

**Figure 4:**
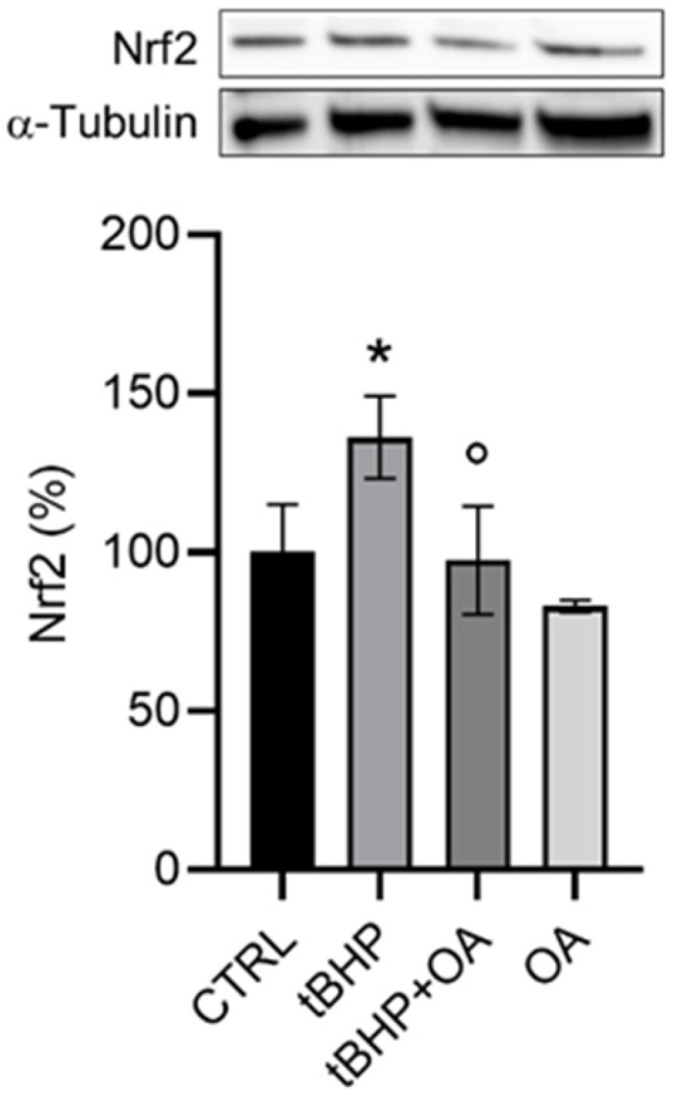
Analysis of Nrf2 expression by Western blot assay in HaCaT cells exposed to the pro-oxidative agent tBHP. HaCaT cells were pre-treated with OA (1.25 µg mL^−1^) for 2 h and then exposed to tBHP (200 µM) for 3 h in serum-free medium. Untreated cells or cells treated with OA or tBHP alone were used as controls. Data are reported as the mean ± SD of three different experiments. Statistical analysis was performed using the Kruskal–Wallis test followed by Conover’s post hoc test (*n* = 3): * *p* < 0.05 vs. CTRL; ° *p* < 0.05 vs. tBHP-treated cells.

Given the cytoprotective action of OA cellular pre-treatment against the oxidative effect of tBHP, the involvement of the Nrf2 signaling pathway was investigated. Data were obtained by Western blot analysis and super-resolution confocal microscopy. Western blot analysis revealed a significant increase in Nrf2 levels in cells treated with tBHP, with an increase of approximately 30% (136 ± 13%) compared with control cells (Figure 4). These data are consistent with literature suggesting the activation of the Nrf2 pathway as a cellular adaptation to oxidative damage [22]. However, cellular pre-treatment with OA did not show any effect on the expression of Nrf2, with levels remaining similar to those of the control (97 ± 17%), as shown in Figure 4.

**Figure 5:**
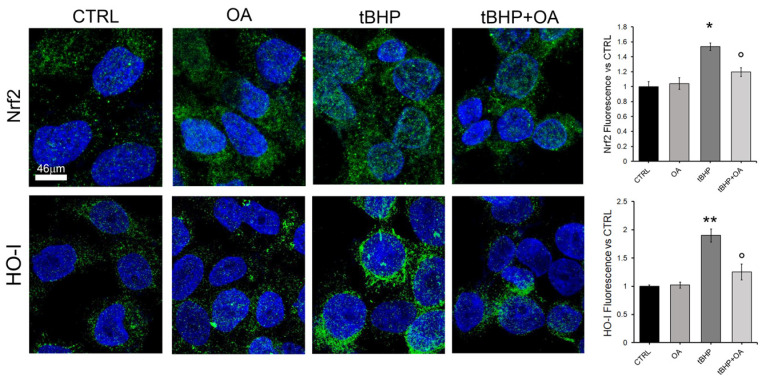
Nrf2 and HO levels in HaCaT cells. Representative confocal images of HaCaT cells pre-treated with OA (1.25 µg mL^−1^) for 2 h and then exposed to tBHP (200 µM) for 3 h in serum-free medium. Nuclei cells were labelled by HOECHST 33342 (blue fluorescence), and Nrf2 and HO were stained with rabbit anti-Nrf2 or anti-HO primary antibodies followed by treatment with Alexa 488-conjugated anti-rabbit secondary antibodies (green fluorescence). Quantification of the green mean fluorescence intensity signals is reported on the right and was performed by ImageJ Fiji software (Version 1.54m). One-way ANOVA test: * *p* < 0.05; ** *p* < 0.01 vs. CTRL; ° *p* < 0.01 vs tBHP. Values are the average of 5 independent experiments± SE.

In addition, the confocal microscopy images shown in Figure 5 provide further confirmation of the cellular mechanisms involved in the oxidative stress response. The images show that upon exposure to tBHP, there is a significant increase in the levels of Nrf2 and HO-1 (Figure 5). Their resultant levels were similar to those in control cells under OA and tBHP+OA conditions, raising intriguing questions about the potential mechanisms by which OA may inhibit Nrf2 activation.

**Figure 6:**
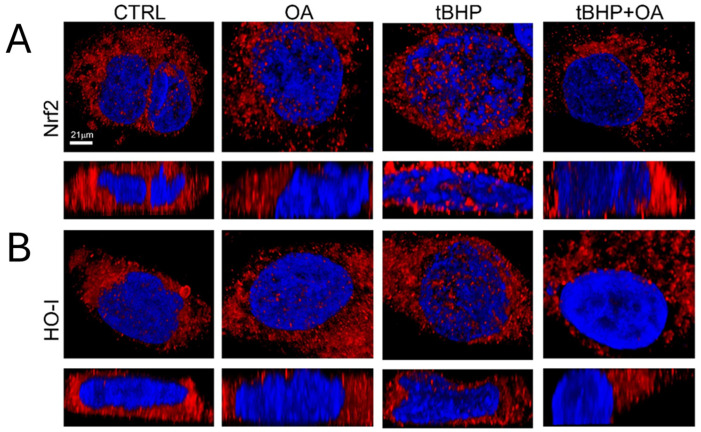
Nrf2 and HO internalization. (**A**,**B**) Representative images from super-resolution confocal microscopy (STED). HaCaT cells were pre-treated with OA (1.25 µg mL^−1^) for 2 h and then exposed to tBHP (200 µM) for 3 h in serum-free medium. Then, HaCaT cells were marked using HOECHST 33342 for nuclei (blue fluorescence), and Nrf2 (**A**) or HO-1 (**B**) was visualized with their specific primary antibody and anti-rabbit Alexa 546 secondary antibodies (red fluorescence). Reconstructions of the z-stack analysis of the cells shown in panels were performed by Leica software (Versions 5.1.0).

HO-1 is a gene regulated by the Nrf2 protein and represents one of the most important cytoprotective molecules in the organism. Due to its ability to degrade free heme, HO-1 plays a fundamental role in protecting cells from damage caused by oxidative stress. It acts not only by reducing the production of ROS but also by promoting cellular repair mechanisms. In this way, HO-1 helps to maintain the redox balance in cells and protects tissues from oxidation-related diseases [21]. Analysis of the nuclear localization of HO-1 using super-resolution confocal microscopy (STED) showed a significant increase in the nuclear localization of Nrf2 (Figure 6A) and HO-1 (Figure 6B) in cells exposed to tBHP. This increase suggests an active engagement of the cellular defense mechanisms against the oxidative stress induced by tBHP, highlighting a crucial role for HO-1 in mitigating cellular damage caused by ROS. In contrast, reduced levels of nuclear HO-1 were observed in cells pre-treated with OA and then exposed to tBHP. This result may indicate that OA pre-treatment inhibits the activation of the Nrf2 pathway, preventing its translocation to the nucleus and the subsequent transcription of HO-1 (Figure 6). This may suggest a different mechanism of action whereby OA does not enhance the oxidative stress response through the classical Nrf2-mediated pathway. It is possible that OA engages other signaling pathways or mechanisms that modulate the cellular response to oxidative stress, possibly through direct antioxidative effects or alternative stress response pathways.

**Figure 7:**
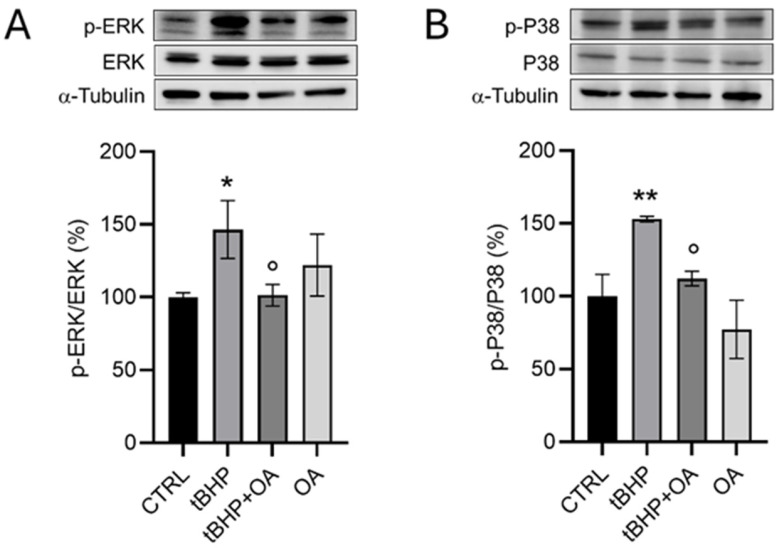
Analysis of (**A**) p-ERK and (**B**) p-p38 as markers of the MAPK signaling pathway in HaCaT cells exposed to the pro-oxidative agent tBHP by Western blot assay. HaCaT cells were pre-treated with OA (1.25 µg mL^−1^) for 2 h and then exposed to tBHP (200 µM) for 3 h in serum-free medium. Untreated cells or cells treated with OA or tBHP alone were used as controls. Data are reported as the mean ± SD of three different experiments. Statistical analysis was performed using the Kruskal–Wallis test followed by Conover’s post hoc test (*n* = 3): * *p* < 0.05; ** *p* < 0.01 vs. CTRL; ° *p* < 0.05 vs. tBHP-treated cells.

These data suggest that OA cellular pre-treatment exerts a cytoprotective action through cellular mechanisms not mediated by the Nrf2 signaling pathway. Analysis of the MAPK pathway further revealed that while tBHP induced an increase in the phosphorylation of ERK (146 ± 19%) and p38 (152 ± 2%), these levels remained unchanged in OA pre-treated cells (Figure 7). These observations lead to the hypothesis that OA exerts its cellular antioxidative protective role independently of the Nrf2 pathway, suggesting an alternative cellular protective mechanism for OA that warrants further investigation.

## 3. Discussion

The aging process is a complex biological phenomenon that manifests itself in cellular dysfunction and an increased vulnerability to diseases, most prominently in the skin. As the body’s largest organ, the skin plays a significant role as a barrier against external environmental threats; however, it is also highly susceptible to age-related changes that compromise its structural integrity and function.

Oxidative stress arises from an imbalance between ROS production and cellular antioxidative defenses. The presence of environmental stressors, such as UV radiation, chemicals, and pollutants, exacerbates cellular ROS generation, leading to harmful consequences for cellular components, including DNA, proteins, and lipids. These dysfunctions in cellular homeostasis contribute not only to the functional decline of skin cells but also to an increased risk of developing a number of skin disorders ranging from dermatitis to more severe conditions such as skin cancer [5,7,23,24].

Aging processes, both the physiological process and those related to the exposure to various environmental factors, have been widely studied in relation to phenomena such as oxidative stress, inflammation, and the glycation of ECM proteins (i.e., collagen). As we have previously demonstrated, OA possesses both anti-inflammatory properties, tested in a cellular model of mouse macrophages exposed to lipopolysaccharide (LPS) [13], and anti-glycation properties, verified in vitro on bovine serum albumin (BSA) and gelatin (as a model for collagen) [14]. This evidence, together with the growing literature emphasizing the benefits of OA [25,26], has sparked our interest in investigating its potential for preventing oxidative stress-induced cell damage in a cellular model of human keratinocytes. Our study investigated exposure to tBHP, a well-known pro-oxidative agent, and the results were promising. Pre-exposure treatments with OA showed effective protection of cell viability and contributed to regulating the production of ROS. These findings support the notion that OA may possess significant antioxidative and cytoprotective properties, providing a relevant contribution to the understanding of the functions and benefits of this molecule in skin health and beyond.

Furthermore, the interaction between NO and OA could have a significant impact on the physiological and pathological course of skin diseases. As discussed in the literature, imbalances in the production or activity of NO can lead to pathological conditions, highlighting the need for the precise regulation of NO levels in tissues. In the skin, NO-associated diseases stem from imbalances in its levels caused by deficiencies or excesses. The dual nature of NO as a beneficial or potentially harmful molecule in the skin is largely determined by the activity of nitric oxide synthase (NOS) enzymes [27,28]. iNOS is an inducible isoform that is normally silent but can be upregulated in response to inflammatory signals or cellular stress [20]. Our data demonstrate that exposure of keratinocytes to tBHP leads to a significant increase both in iNOS expression and, consequently, to an increase in NO levels. In the skin, the iNOS-derived NO plays a crucial role in immune and inflammatory responses, and aberrant iNOS activity and NO production have been implicated in various skin pathologies [20]. A promising aspect highlighted by our research is that pre-treatment with OA not only counteracts intracellular ROS production but also reduces iNOS expression in HaCaT cells, suggesting some modulation of NO levels. This mechanism of action of OA in cell protection may represent an important therapeutic approach for the treatment of skin diseases, in which oxidation plays a crucial role. Additionally, OA’s ability to reduce the excessive production of NO by iNOS could prove to be an interesting strategy to prevent the cellular damage resulting from an imbalance in NO homeostasis.

In order to understand the mechanisms by which OA confers cytoprotection against oxidative stress on keratinocytes, this study also investigated the possible involvement of the Nrf2 pathway. Indeed, the Nrf2 signaling pathway is recognized as the main regulator of the cellular antioxidative response, activating the expression of over 200 genes involved in the defense against oxidative stress. Under homeostatic cellular conditions, Nrf2 levels are kept low, but in response to pro-oxidative stimuli, Nrf2 accumulates and translocates from the cytosol to the nucleus, activating target genes such as HO-1 [9]. This study highlights the role of the pro-oxidative stimulus tBHP in inducing intracellular ROS production and the subsequent activation of the Nrf2 signaling pathway in HaCaT keratinocytes. The results clearly show an increase in Nrf2 levels, accompanied by its translocation to the nucleus, and a subsequent increase in HO-1 expression at the nuclear level. These findings are consistent with what has been reported in the literature, where it has been documented that situations of intracellular cellular oxidation can activate the Nrf2 pathway as an adaptive response to restore redox balance and prevent ROS-induced toxicity [29].

In addition, an interesting connection with the mitogen-activated kinase (MAPK) system emerges: the MAPK signaling pathway was found to be activated in response to the pro-oxidative tBHP exposure, confirming what has already been reported in the literature regarding the response of MAPKs to various stimuli, including oxidative stress. This activation should not be underestimated, as MAPKs play a significant role in Nrf2 induction. Therefore, the interaction between MAPKs and Nrf2 represents an important synergy in cell signaling pathways, enabling cells to adapt and respond to adverse oxidative conditions [30].

An interesting aspect of our study emerged with the cell pre-treatment with OA, which was shown to prevent the induction of intracellular ROS production by tBHP exposure. Our results suggest that the absence of a significant increase in intracellular ROS in OA pre-treated cells, even when the cells are exposed to tBHP, indicates that the Nrf2 pathway is not activated as expected. Cells pre-treated with OA show expression levels of Nrf2 and HO-1 comparable to those in control cells, suggesting a potential stabilizing and cytoprotective effect of OA against oxidative stress. This OA cytoprotective role is confirmed by the reduced levels of intracellular ROS in OA pre-treated cells when exposed to the pro-oxidative tBHP.

Although Nrf2 plays a central role in the cellular defense against oxidative stress by regulating the expression of various antioxidative and detoxifying enzymes, there are also pathways that contribute to cellular protection and the stress response that are independent of Nrf2. The literature suggests that OA may exert protective effects through Nrf2-independent pathways, i.e., by regulating the expression of a 70 kDa heat shock protein (Hsp70) [31]. In fact, heat shock proteins, a group of proteins that help protect cells from stress, can be induced by various stress signals and are regulated by different pathways independently of Nrf2 [32].

There are also several other mechanisms capable of protecting cells from oxidative stress independently of Nrf2 activation. Some antioxidative enzymes can be regulated by transcription factors other than Nrf2 [33]. For instance, activation of specific signaling pathways can lead to an upregulation of these enzymes without the direct involvement of Nrf2.

Nuclear factor kappa-light-chain-enhancer of activated B cells (NF-κB) is also a critical transcription factor involved in the inflammatory response. It can regulate genes related to oxidative stress and inflammation, providing a protective response independently of Nrf2, as reported in the literature [34].

However, further investigations are needed to elucidate the specific mechanisms underlying the OA effects.

Therefore, the antioxidative properties of OA in human keratinocyte cells exposed to a stress factor, along with its already demonstrated anti-inflammatory and anti-glycation potential, make OA an excellent candidate for further studies on skin prevention and protection in the aging process and skin care [35]. Indeed, this molecule could play a crucial role in maintaining skin health by counteracting the harmful effects of free radicals and inflammatory processes, thereby contributing to the improvement in skin appearance and functionality over time. Due to its anti-inflammatory properties [36], OA could be used against various inflammatory skin diseases, such as atopic dermatitis and psoriasis [37,38]. Furthermore, its role as a modulator of lipid metabolism presents interesting opportunities for pharmaceutical development [39,40]. For instance, OA could be investigated as a potential agent to improve the skin’s barrier function, increase hydration, and deliver active compounds more effectively in topical formulations.

Looking forward, the implications of OA in skin care and pharmaceutical development are significant. As the demand for natural and effective skin care solutions increases, the incorporation of OA into formulations could provide a competitive advantage.

Furthermore, despite the known poor aqueous solubility and limited bioavailability of OA, numerous studies have explored various approaches and advances in OA-based drug delivery systems, aiming to improve the biopharmaceutical characteristics and overall efficacy in different therapeutic contexts [36]. These efforts are aimed at overcoming the challenges associated with OA formulation to make it more accessible and usable in clinical settings.

In this regard, future research could open up new perspectives for the use of OA, not only in therapeutic contexts but also in cosmetic formulations. In fact, the incorporation of OA into cosmetic products could prove particularly beneficial for slowing down the signs of aging, thanks to its antioxidative and anti-inflammatory properties. Continuous innovation in formulation technologies could facilitate the absorption and effectiveness of OA, making it a key ingredient for the development of innovative and multifunctional products.

Finally, the study of olive-leaf-derived OA represents a unique intersection between health benefits and environmental sustainability. As the olive tree industry produces significant amounts of waste, mainly in the form of olive leaves, the opportunity to recycle this by-product into a valuable health compound underlines the importance of moving toward a circular bioeconomy. This approach not only aims to mitigate the environmental impact of waste production but also contributes to the development of sustainable health products that respond to the growing consumer demand for natural and effective alternatives to synthetic drugs.

The bioactive properties of OA deserve extensive discussion, especially in light of its antioxidative potential.

In this context, skin-related disorders are increasingly recognized as being influenced by oxidative damage, and the protective qualities of OA further amplify its relevance in the dermatological and skin care field.

## 4. Materials and Methods

### 4.1. Materials and Reagents

Oleanolic acid (OA) was sourced from Natac Biotech SL, Getafe, Madrid, Spain, and utilized at specified concentrations for subsequent experiments. OA is of natural origin, obtained by ethanol extraction from the leaves of *Olea europaea* L.; it has a purity of 97%, defined by HPLC-DAD analysis using an OA standard with purity 99%. For cell culture and cell-based in vitro experiments, all necessary reagents were procured from Merck KGaA (Darmstadt, Germany), including Dulbecco’s Modified Eagle Medium (DMEM) culture medium, antibiotics (penicillin and streptomycin), L-glutamine, Fetal Bovine Serum (FBS), trypsin-EDTA solution, and Phosphate-Buffered Saline (PBS). To evaluate cell viability and cytotoxicity, 1-(4,5-dimethylthiazol-2-yl)-3,5-diphenyl formazan (MTT) was used. The intracellular reactive oxygen species (ROS) levels were quantified using the 2′,7′-dichlorofluorescin diacetate (DCFDA) fluorescent probe. The determination of nitrite levels in culture supernatants was conducted using Griess reagent. Primary and secondary antibodies used for the Western blot assays are listed in Table 1. Additionally, sterile disposable plasticware, including culture plates and pipette tips, was obtained from Sarstedt (Verona, Italy). All reagents were utilized according to the manufacturer’s instructions, maintaining appropriate sterile techniques to ensure experimental integrity.

### 4.2. Cell Line and Culture Conditions

Human keratinocytes (HaCaT) cells, which are spontaneously transformed keratinocytes derived from histologically normal skin, were obtained from Cell Line Service (CLS, catalog number 300493). The cells were cultured in Dulbecco’s Modified Eagle Medium (DMEM) supplemented with 2 mM L-glutamine, 100 µg mL^−1^ streptomycin, 100 U/mL penicillin, and 10% fetal bovine serum (FBS), referred to as complete medium. The cell culture was maintained at 37 °C in a humidified atmosphere containing 5% CO_2_. Upon reaching 80–90% confluence, the cells were detached using a solution comprising 0.025% EDTA and 0.5 mM trypsin. After detachment, the cells were diluted to the appropriate concentration for further experiments. All subsequent in vitro assays were performed using serum-free medium to minimize variability due to serum components.

### 4.3. MTT Assay

To assess cell viability, HaCaT cells were cultured in 96-well plates at a density of 1 × 10^4^ cells per well and allowed to adhere overnight. Cells were pre-treated with different concentrations of OA (0–10 µg mL^−1^) for 24 h to assess the dose-dependent effect of OA on cell viability over time. In the following experiments, pre-treatment with OA was carried out at the lowest non-toxic dose (1.25 µg mL^−1^) for 2 h. After OA pre-treatment, the cells were treated with tert-butyl hydroperoxide (tBHP) at a concentration of 200 µM for 3 h in serum-free DMEM. The concentration of tBHP was chosen in accordance with the literature [41]. Control groups included untreated cells, cells treated only with OA, and cells treated only with tBHP. Next, the culture medium was removed, and 100 µL of MTT solution (0.5 mg mL^−1^ in PBS) was added to each well. The plates were incubated in the dark at 37 °C for 1 h to allow metabolically active cells to reduce MTT to formazan crystals. Following incubation, formazan crystals were dissolved by adding 100 µL of dimethyl sulfoxide (DMSO) to each well with gentle shaking to ensure complete dissolution. The optical density was measured at 595 nm using a microplate reader to determine the cell viability, and the data were expressed as a percentage of untreated control cells (CTRL).

### 4.4. Intracellular ROS Detection

Intracellular ROS levels were quantified using the cell-permeable fluorescent probe 2,7-dichlorodihydrofluorescein diacetate (DCFDA). HaCaT cells were seeded on a 96-well plate at a density of 1 × 10^4^ cells per well in complete medium and incubated overnight. The cells were pre-treated with 1.25 µg mL^−1^ of OA for 2 h and then treated with tBHP at a concentration of 200 µM for 3 h in serum-free DMEM. After treatment, 10 µM of DCFDA probe in PBS was added to each well and incubated for 1 h at 37 °C in the dark. Fluorescence was measured at excitation and emission wavelengths of 485 and 538 nm, respectively, using a Biotek Synergy 1H plate reader (Agilent Technologies, Santa Clara, CA, USA). The ROS levels were normalized to cell viability, and the data were presented as a percentage relative to untreated control cells (CTRL).

### 4.5. Nitric Oxide Determination

HaCaT cells were seeded at a density of 4 × 10^4^ cells per well in a 96-well plate and incubated for 24 h. Following the same experimental pre-treatment procedure with 1.25 µg mL^−1^ of OA for 2 h and exposure to tBHP at 200 µM for 3 h in serum-free DMEM, nitrite levels in the culture medium were measured to determine the nitric oxide (NO) production using the Griess reaction. Briefly, 50 µL of cell culture medium from each treatment was mixed with an equal volume of Griess reagent and incubated at room temperature for 15 min. Absorbance at 540 nm was recorded using an iMARK microplate reader. The concentration of NO was determined using sodium nitrite as a reference within a range of 0–50 µM. The data were normalized to cell viability and expressed as a percentage with respect to untreated control cells (CTRL).

### 4.6. Western Blot Assay

HaCaT cells (15 × 10^4^ cells per well) were cultured in 6-well plates for 24 h. After undergoing pre-treatment with OA (1.25 µg mL^−1^ for 2 h), the cells were exposed to tBHP at 200 µM for 3 h in serum-free DMEM. The cell lysates were prepared using Laemmli buffer containing 62.5 mM Tris-HCl (pH 6.8), 10% (*w*/*v*) SDS, and 25% (*w*/*v*) glycerol. Cell lysates were then centrifuged at 4 °C for 1 min at 12,000× *g*. The total protein concentration was measured using the BCA protein assay. A corresponding equal-protein aliquot (30 µg protein) from each sample was combined with 5% (*v*/*v*) β-mercaptoethanol and bromophenol blue and heated at 95 °C for 5 min. Protein samples were electrophoretically separated on 12% SDS–polyacrylamide gels and then transferred onto PVDF membranes (0.45 µm). After blocking with a BSA solution (5% (*w*/*v*) BSA in 0.1% (*v*/*v*) PBS-Tween^®^-20), membranes were incubated overnight at 4 °C with appropriately diluted primary antibodies (listed in Table 1). Following three washes with a 0.1% (*v*/*v*) PBS-Tween^®^-20 solution, HRP-linked secondary antibodies (goat anti-rabbit IgG and goat anti-mouse IgG, both at 1:10,000 dilution; Invitrogen, Waltham, MA, USA) were added for 1 h at room temperature. After three additional washes with a 0.5% (*v*/*v*) PBS-Tween^®^-20 solution, Clarity Western ECL solution was applied for protein band detection using the Amersham^TM^ 600 Imager imaging system (GE Healthcare Life Science, Pittsburgh, PA, USA). Densitometric analysis of protein bands was conducted using Quantity One software (version 4.6.6, Bio-Rad, Milan, Italy).

### 4.7. Super-Resolution Confocal Microscopy

STED xyz images were acquired by using an SP8 STED 3X confocal microscope (Leica Microsystems, Mannheim, Germany). Cell nuclei were stained with HOECHST 33342 for 30 min at 37 °C. HO or Nrf2 were detected with 1:500 diluted mouse monoclonal primary antibodies and 1:500 Alexa Fluor 546-conjugated anti-mouse secondary antibody (A-31555, Thermo Fisher Scientific, Waltham, MA, USA). Fluoromount-G™ (00-4958-02, Fisher Scientific) was used as the mounting medium. Images were acquired with a Leica HC PL APO CS2 100×/1.40 oil STED White objective. Gated pulsed-STED was applied to the Alexa Fluor 546 fluorophore. Collected images were analyzed with Leica Application Suite X (LAS X) software (Leica) (Versions 5.1.0) to generate 3D reconstructions.

### 4.8. Confocal Microscopy

Subconfluent HaCaT cells grown on glass coverslips were treated as previously reported. Cell nuclei were stained with HOECHST 33342 for 30 min at 37 °C. HO and Nrf2 proteins were stained with mouse monoclonal anti-HO antibodies (1:500) and with anti-Nrf2 antibodies (1:500) followed by anti-mouse Alexa Fluor 488-conjugated secondary antibodies (green channel). Cell fluorescence was imaged using a confocal Leica TCS SP8 scanning microscope (Leica, Mannheim, DE, USA). The observations were performed using a Leica HC PL Apo CS2 X63-oil immersion objective. Images and signal fluorescence were composed and analyzed by Image J Fiji software (Version 1.54m).

### 4.9. Statistics

All data are expressed as the mean ± standard deviation (SD) of independent experiments, unless otherwise indicated. Statistical analysis was performed using one-way ANOVA followed by the post hoc Tukey HSD test to assess the differences among groups for normally distributed data. For Western blot analysis, differences between normalized intensity signals were evaluated using the Kruskal–Wallis test followed by Conover’s post hoc test, which is appropriate for non-parametric data. Statistical significance was defined as *p* ≤ 0.05.

To ensure the reproducibility and reliability of the results, all experiments were conducted in triplicate. Statistical analyses were carried out using GraphPad Prism, version 8.4.3.

## 5. Conclusions

In conclusion, our pilot study on the effects of OA against tBHP-induced oxidative stress in HaCaT cells highlights the potential protective role of this bioactive compound in skin health and aging. The results underline the ability of OA to prevent the formation of intracellular ROS and to reduce the expression of iNOS and NO release in keratinocytes, thereby counteracting oxidative stress and potential cellular damage. These findings, together with the anti-inflammatory and anti-glycation properties we have previously demonstrated, indicate the promising potential of OA in mitigating oxidative damage and preserving the structural integrity of skin. Furthermore, this study suggests that OA may provide protection against oxidative stress in keratinocytes through a mechanism independent of the Nrf2 and MAPK signaling pathways. The possibility of extracting OA from olive leaves also has significant implications for both human health and environmental sustainability, which is in line with the growing movement toward circular bioeconomy practices. In an effort to promote an environment beneficial for both human health and ecological balance, OA is emerging as a key component in achieving these dual objectives. The capacity to modulate cellular responses in the context of oxidative stress with natural compounds could have considerable implications for enhancing skin health and developing treatments for oxidation-related diseases. Future research could greatly enhance our understanding of skin health and aging, elucidating the specific pathways through which OA exerts its effects. Our results emphasize the need for interdisciplinary approaches that integrate biochemistry, human health, and environmental sciences, ultimately guiding future research efforts. This holistic perspective represents a significant step forward in promoting a healthier population and a sustainable planet.

## Figures and Tables

**Table 1:** List of primary antibodies used in the Western blot assay.

Primary Antibody	Dilution	Isotype	Source
iNOS	1:1000	Rabbit IgG	Cell Signaling
Nrf2	1:1000	Mouse IgG	Santa Cruz
p-p38	1:1000	Mouse IgG	Santa Cruz
p38	1:1000	Mouse IgG	Santa Cruz
p-ERK1/2	1:2000	Rabbit IgG	Cell Signaling

## Data Availability

Data is contained within the article.
